# Impact of job adjustment, pain location and exercise on sick leave due to lumbopelvic pain in pregnancy: a longitudinal study

**DOI:** 10.1080/02813432.2019.1608058

**Published:** 2019-05-06

**Authors:** Signe N. Stafne, Nina K. Vøllestad, Siv Mørkved, Kjell Å. Salvesen, Hilde Stendal Robinson

**Affiliations:** a Department of Public Health and Nursing, Norwegian University of Science and Technology (NTNU), Trondheim, Norway;; b Clinical Services, St. Olavs Hospital, Trondheim University Hospital, Trondheim, Norway;; c Department of Health Sciences, Institute of Health and Society, University of Oslo, Norway;; d Research Department, St. Olavs Hospital, Trondheim University Hospital, Trondheim, Norway;; e Department of Obstetrics and Gynecology, St. Olavs Hospital, Trondheim University Hospital, Trondheim, Norway;; f Institute of clinical and molecular medicine, Norwegian University of Science and Technology (NTNU), Trondheim, Norway

**Keywords:** Sick leave, pregnancy, lumbopelvic pain, exercise, job adjustment

## Abstract

**Objective:** To identify factors associated with sick leave due to lumbopelvic pain (LPP) in pregnancy.

**Design:** Prospective cohort study using participants from a randomized controlled trial (RCT) designed to study the effect of exercise during pregnancy on pregnancy related diseases.

**Setting:** St. Olavs Hospital, Trondheim University Hospital and Stavanger University Hospital, April 2007 to December 2009.

**Subjects:** Healthy pregnant women.

**Main outcome measures:** Self-reported sick leave due to LPP in late pregnancy (gestation week 32–36).

**Results:** In total, 532/716 (74%) women reported LPP at 32–36 weeks of pregnancy, and 197/716 (28%) reported sick leave due to LPP. Not receiving job adjustments when needed (Odds ratio, OR with 95% confidence interval, CI, was 3.0 (1.7–5.4)) and having any pain in the pelvic girdle versus no pain (OR 2.7 (1.3–5.6), OR 2.7 (1.4–5.2) and OR 2.2 (1.04–4.8)) for anterior, posterior and combined anterior and posterior pain in the pelvis respectively, were associated with sick leave due to LPP in late pregnancy. Also higher disability, sick listed due to LPP at inclusion and lower education, were significant explanatory variables. There was a trend of reduced risk for sick leave due to LPP when allocated to the exercise group in the original RCT (OR 0.7 (0.4–1.0)).

**Conclusion:** Facilitating job adjustments when required might keep more pregnant women in employment. Furthermore, pain locations in pelvic area, disability, lower education and being sick listed due to LPP in mid pregnancy are important risk factors for sick leave in late pregnancy.

**Key points**
Current awareness:
More than half of pregnant women are on sick leave during pregnancy and the most frequently reported cause is lumbopelvic pain.
*This paper adds:*
  - Inability to make job adjustments, pain locations in pelvic area, disability and lower education level were the most important risk factors for sick leave in late pregnancy. Facilitating early job adjustment might be a precaution to keep more pregnant women in work. Allocation to an exercise group tended to reduce the risk of sick leave in late pregnancy.**Registration number:** Clinical trial gov (NCT 00476567).

## Introduction

Three out of four women in fertile age (25–54 years) in the European Union are employed [1]. Pregnancy is considered a normal physiological condition, but may present physical challenges compromising a woman’s ability to work. European Union legislation requires employers to assess health and safety risks to pregnant workers and, where necessary, to temporarily adjust working conditions and/or working hours [2]. Pregnancy related sick leave introduces large economic costs for society and personal distress for individual women. How women can remain at work during pregnancy is important for individuals and society.

One recent study, comparing 12 European countries and including 9483 women, found that 50.6% had been on sick leave at some point during pregnancy [3]. The rates of sick leave varied greatly within Europe, ranging from 31.7% in Sweden to 71.3% in Poland [3]. Duration of sick leave shows large variation, with one Norwegian study reporting 50% of pregnant women being off work between 4 and 16 weeks [4].

The most frequently reported cause of sick leave in pregnant women is lumbopelvic pain (LPP) [3,4]. LPP is used as a collective term for low back and/or pelvic girdle pain, and studies have shown prevalence around 50% in late pregnancy [5–8]. LPP may restrict weight-bearing activities, interfere with ordinary daily activities [9] and reduce quality of life [5]. Considerable difficulties with activities such as housekeeping, walking, working and sexual life have been reported [10,11]. Furthermore, LPP may influence the women’s workability and job adjustments can be important to keep women at work through pregnancy.

The aim of the present study was to identify factors associated with being on sick leave due to LPP in late pregnancy.

## Material and methods

We conducted a prospective cohort study using participants of a randomized controlled trial (RCT), designed to study the effect of exercise during pregnancy on pregnancy related diseases [12].

Pregnant women booked for routine ultrasound scans at St. Olavs Hospital, Trondheim University Hospital and Stavanger University Hospital in Norway were invited to participate in a two-armed, two-center RCT comparing a 12-week regular exercise program with standard antenatal care [12]. The exercise program included exercises to strengthen large muscle groups, body awareness and lifting techniques. Women in the exercise group received unstructured verbal information from the physiotherapists in charge of the exercise groups, about anatomy, pregnancy related changes and ergonomics. Both groups received written information about LPP. Data were collected at inclusion (gestation week 18–22) and after the intervention period (gestation week 32–36). Inclusion criteria were age over 18 years and being pregnant with a singleton live fetus. Exclusion criteria were high-risk pregnancies and/or diseases that could interfere with participation. For practical reasons women living too far from the hospitals (more than 30 minutes drive away) were excluded (not able to attend weekly training groups) [12].

### Outcome variable

The principal outcome of the present study was sick leave due to LPP in late pregnancy. Participants answered a questionnaire at inclusion (gestation week 18–22) and again at gestation week 32–36. We used data collected at gestation week 32–36, in addition to some descriptive data from the time of inclusion. Data on sick leave were self-reported. Women were asked if, and for what reason, they had been on sick leave during pregnancy. Multiple answers were allowed, including low back pain (LBP), pelvic girdle pain (PGP), contractions, nausea/vomiting, sleep problems, fatigue, blood pressure, and “other”. Women including LBP and/or PGP as reason for being sick listed were defined as cases. Women not sick listed due to LBP and/or PGP were defined as controls.

### Explanatory variables

Potential explanatory variables were pain location, occupational exposures (work schedule, work tempo and working walking/standing), job adjustment, disability, fear–avoidance beliefs, sick listed at inclusion, parity, age, BMI, education and intervention group allocation. Presence of LPP was based on one question: “*Do you have pain in the pelvic and/or lumbar area?” (Yes/No).* If the women reported PGP, pain location was assessed with the following categories; (1) anterior pelvic pain (symphyseal pain), (2) unilateral posterior pelvic pain, (3) bilateral posterior pelvic pain, (4) combined anterior and unilateral posterior pelvic pain, and (5) combined anterior and bilateral posterior pelvic pain. In the analysis unilateral and bilateral posterior pain were merged into one variable; “posterior pain” and combined anterior and posterior unilateral/bilateral pelvic pain were merged into “combined anterior and posterior pelvic pain”. Previously, women with combined pain in the anterior and posterior pelvis have shown worse prognosis and more disability [13,14]. Women reporting to have pain in the pelvic and/or lumbar area, but no location in the pelvis, were categorized as having LBP. Only five women who presented with no pain in early pregnancy were on sick leave due to LPP in late pregnancy. Thus for analyzing purposes, we merged the groups reporting no pain with the LBP group (no pain/LBP).

Women were asked if their work situation had been adjusted to accommodate for their pregnancy. Answer alternatives were yes/no, as well as whether this was not necessary or not possible. Disability was measured using the Disability Rating Index (DRI). DRI contains 12 questions about the ability to perform activities of daily living. Each question is scored on a 100 mm visual analogue scale (VAS) from 0 = “ability to perform activity without restriction” to 100 = “inability to perform activity” [15]. DRI is calculated as the mean of the 12 scores.

Modified Fear-Avoidance Beliefs Questionnaire (mFABQ) consists of the four questions from the physical activity part of the fear-avoidance beliefs questionnaire [16]. Ratings were made on Likert scales (0 to 6) ranging from strongly disagree (0) to strongly agree (6). The four scores were added, and the sum score ranged from 0 to 24 with high scores showing stronger fear-avoidance beliefs.

Questions regarding job characteristics and work environment were included at baseline. These contained work schedule (regular daytime, regular afternoon/evening, regular night-time, shift/rota, temporary work/substitute), working involving walking/standing (daily >50%, daily ≤50%, periodically, seldom/never) and ability to decide on working tasks and tempo (daily >50%, daily ≤50%, periodically, seldom/never). Women were weighed with light clothing at inclusion, and height, parity and age were self-reported.

Study procedures followed the Helsinki declaration. All women received written information, and signed informed consent forms. Participants did not receive any financially compensation. The Regional Committees for Medical and Health Research Ethics approved the study (REK 4.2007.81), and the trial was registered in Clinical trial gov (NCT 00476567).

## Statistical analyses

Analyses were performed using SPSS statistical package version 22 (IBM Corp., New York, NY). Descriptive data are presented as mean with standard deviation (SD) and frequencies (%) as appropriate. Associations between explanatory variables and the outcome variable were studied by Spearman rank correlation coefficients. Odds ratios (OR) with 95% Confidence Intervals (95% CI) of sick leave due to LPP in late pregnancy for potential explanatory variables were estimated in univariate and multivariable logistic regression models. A 5% level of significance was used.

## Results

**Figure 1.**
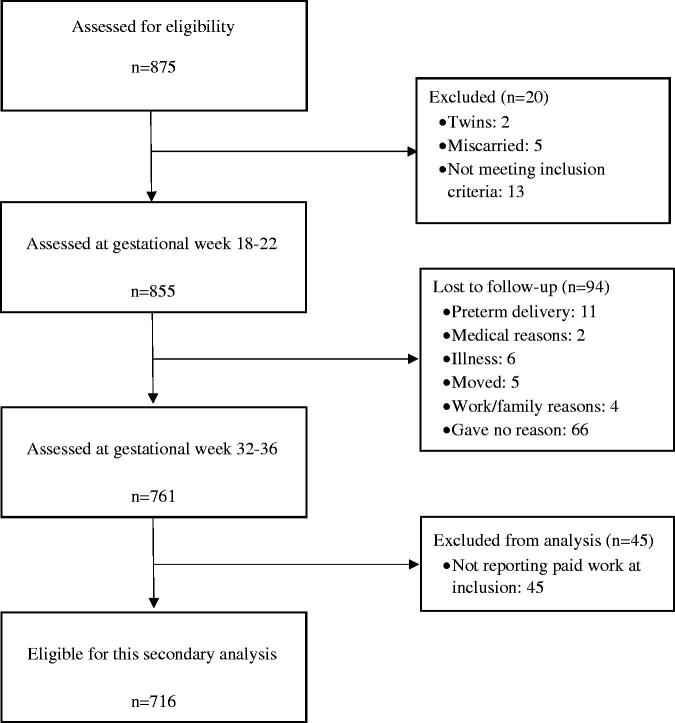
Flow chart of study participants.

In total, 855 pregnant women were included in the original study [12], and 716 had complete data at follow up (Figure 1). Baseline characteristics of the participants are shown in Table 1 and did not differ from the original study population.

**Table 1.** Baseline characteristics at study entry (18–22 weeks of pregnancy) of all women included in the original RCT (*N* = 855) and women included in the present cohort (*N* = 716; in paid work and assessed at follow-up 32–36 weeks of pregnancy).

	Original study population (RCT) *N* = 855		Study population (Cohort) *N* = 716	
	Frequency (%)	Mean (SD)	Frequency (%)	Mean (SD)
Age		30.4 (4.3)		30.7 (4.1)
BMI		24.8 (3.2)		24.7 (3.1)
Parity				
0	486 (57)		410 (57)	
1	254 (30)		212 (30)	
≥2	115 (13)		94 (13)	
Self-reported LPP	502 (59)		420 (59)	
Pain location				
No LPP	350 (41)		294 (41)	
Low back pain	122 (14)		100 (14)	
Anterior pelvic pain	42 (5)		39 (5)	
Posterior pelvic pain	264 (31)		220 (31)	
Combined anterior and posterior pelvic pain	74 (9)		61 (9)	
DRI sum score^a^		16.2 (15.2)		16.1 (15.3)
mFABQ		8.6 (4.0)		8.6 (4.0)
On sick leave due to LPP	37 (4)		31 (4)	
Education				
≤13 years school attendance	95 (11)		69 (10)	
≤4 years university	331 (39)		279 (39)	
>4 years university	429 (50)		368 (51)	
Paid work	798 (93)		716 (100)	
Working hours^b^				
Regular daytime	567 (71)		511 (71)	
Regular afternoon/evening	17 (2)		16 (2)	
Regular night-time	15 (2)		12 (2)	
Shift/rota	174 (22)		160 (22)	
Temporary work/substitute	22 (3)		14 (2)	
Working walking/standing^b^				
Daily >50%	325 (41)		285 (40)	
Daily ≤50%	132 (17)		122 (17)	
Periodically	89 (11)		82 (12)	
Seldom/never	248 (31)		223 (31)	
Able to decide working tasks and tempo myself^b^				
Daily >50%	274 (34)		247 (35)	
Daily ≤50%	76 (10)		70 (10)	
Periodically	215 (27)		193 (27)	
Seldom/never	228 (29)		201 (28)	

BMI: Body Mass Index; LPP: lumbopelvic pain; DRI: Disability Rating Index; mFABQ: modified Fear-Avoidance Beliefs Questionnaire.

a Calculated for women reporting LPP.

b Calculated for women reporting paid work.

A total of 532 (74%) of 716 women reported LPP at 32–36 weeks of pregnancy, and 197 (28%) of 716 were on sick leave due to LPP between inclusion and follow-up. Correlation coefficients between sick leave due to LPP and explanatory variables are shown in Table 2. The correlation between the explanatory variables varied from −0.257 to 0.569 and showed no multicolinearity (data not shown). In the original RCT, there was no difference between the intervention and control groups regarding sick leave for any other reasons than LPP (232 (52%) of 394 in intervention group and 217 (48%) of 365 in control group, *p* = 0.873).

**Table 2.** Correlation between sick leave due to LPP in late pregnancy and potential risk factors.

	Sick leave due to LPP in late pregnancy
DRI score at gestation week 32–36 (0–100)	0.536**
Sick listed due to LPP at inclusion	0.330**
Allocated to the exercise group	−0.084*
Education	0.105*
Received job adjustment	0.280**
Pain location (no LPP, low back pain, anterior pelvic pain, posterior pelvic pain, combined anterior and posterior pelvic pain)	0.375**
BMI	0.090*
Fear avoidance beliefs	0.100*
Work schedule	0.113*
Work tempo	0.085*
Working walking/standing	0.144*
Number of children	0.106**
Age	−0.037

LPP: lumbopelvic pain; DRI: Disability Rating Index; BMI: Body Mass Index.

Spearman’s correlation coefficient; **0.001<*p* ≤ 0.01, *0.01<*p* < 0.05.

Mean (SD) DRI for women reporting LPP was 28.1 (±20.1). In the multivariable logistic model, higher DRI scores were associated with sick leave due to LPP in late pregnancy (Odds Ratio, OR 1.07 with 95% Confidence interval CI (1.05, 1.08)). Sick leave due to LPP in late pregnancy was associated with having any form of pain in the pelvic girdle versus no pain (OR 2.7 (1.3, 5.6), OR 2.7 (1.4, 5.2) and OR 2.2 (1.04, 4.8)) for anterior, posterior and combined anterior and posterior pain, respectively. Mean DRI score (SD) was, however, higher for women with combined anterior and posterior pain (41.2 (21.8)) than for women with posterior pain (24.9 (18.7)), women with anterior pain (25.4 (16.5)) and women with no pain (9.9 (12.9)). Hence, we have chosen to present two multivariable models, one including DRI (model 1) and one without DRI (model 2). The OR’s for being on sick leave due to LPP increased for all pelvic pain locations in model 2. Being sick listed due to LPP at inclusion was also associated with sick leave due to LPP in late pregnancy (OR 31.1 (2.7, 351.1)). Women with >4 years education at university were less likely to be sick listed due to LPP compared with women reporting ≤4 years education at university (OR 2.4 (1.5, 3.9)) or upper secondary school (OR 2.3 (1.1, 4.7)). Reporting “*yes, received job adjustments”* or “*no, it was not possible”* was compared to *“job adjustments were not necessary”* and both were associated with sick leave due to LPP (OR 1.6 (0.9, 2.6) and OR 3.0 (1.7, 5.4)). There was a trend of reduced risk for sick leave due to LPP when allocated to the exercise group in the original RCT (OR 0.7 (0.4, 1.0)) (Table 3).

**Table 3.** Crude and adjusted OR with 95% confidence interval for women, in paid work, being on sick leave due to LPP in late pregnancy, *n* = 716.

	Sick leave due to LPP in late pregnancy		Crude values		Adjusted Model 1		Adjusted Model 2	
	Yes	No	OR (95% CI)	*p* Value	OR (95% CI)	*p* Value	OR (95% CI)	*p* Value
Sum DRI score at pregnancy week 32–36	41.9 (20.1)	20.3 (15.4)	1.07 (1.06, 1.09)	<0.001	1.07 (1.05, 1.08)	<0.001	–	–
Sick listed due to LPP at inclusion								
No	30 (15)	1 (0.2)	Reference	<0.001	Reference	0.005	Reference	<0.001
Yes	168 (85)	559 (99.8)	93.1 (12.5, 687.6)		31.1 (2.7, 351.1)		46.6 (5.9, 369.8)	
Allocated to the exercise group in original RCT								
No	108 (55)	236 (45)	Reference	0.026	Reference	0.058	Reference	0.064
Yes	89 (45)	283 (55)	0.69 (0.49, 0.96)		0.7 (0.4, 1.0)		0.7 (0.5, 1.0)	
Education								
More than 4 years at University	72 (37)	296 (57)	Reference	0.001	Reference	0.001	Reference	0.002
4 or less years at University	94 (48)	185 (36)	2.1 (1.5, 3.0)		2.4 (1.5, 3.9)		1.9 (1.3, 2.9)	
Upper secondary school	31 (16)	38 (7)	3.3 (2.0, 5.8)		2.3 (1.1, 4.7)		3.9 (2.3, 6.6)	
Received job adjustment								
“Not necessary”	45 (24)	256 (50)	Reference	<0.001	Reference	0.001	Reference	<0.001
“Yes, got adjustments”	77 (41)	183 (36)	2.4 (1.6, 3.6)		1.6 (0.9, 2.6)		1.8 (1.2, 2.9)	
“No, it was not possible”	68 (36)	71 (14)	5.4 (3.4, 8.6)		3.0 (1.7, 5.4)		3.9 (2.3, 6.6)	
Pain location in late pregnancy								
No pain/LBP	20 (10)	241 (47)	Reference	<0.001	Reference	0.023	Reference	<0.001
Anterior pelvic pain	37 (19)	80 (16)	5.6 (3.1, 10.2)		2.7 (1.3, 5.6)		6.2 (3.2, 11.9)	
Posterior pelvic pain	73 (37)	135 (26)	6.5 (3.8, 11.2)		2.7 (1.4, 5.2)		5.2 (2.9, 9.4)	
Combined anterior and posterior pelvic pain	67 (34)	61 (12)	13.2 (7.5, 23.1)		2.2 (1.04, 4.8)		11.0 (5.8, 20.9)	

Values are presented as mean (±SD) *or n* (%).LPP: lumbopelvic pain; DRI: Disability Rating Index; LBP: low back pain. Variables included in the bivariate regression analysis, but not significantly associated with sick leave due to LPP in late pregnancy: parity, BMI, mFABQ, work schedule, working walking/standingand whether women were able to decide working tasks and tempo themselves.

Variables not significantly associated with sick leave due to LPP were: parity, BMI, mFABQ, work schedule, working walking/standing and ability to decide working tasks and tempo (Table 3).

## Discussion

### Statement of principal findings

In this study women in no need for job adjustments were less likely to be on sick leave due to LPP compared to women having received job adjustments and women reporting that adjustments were not possible. Furthermore, more than one pain location in the pelvic girdle and higher disability scores increased the risk, whereas higher education reduced the risk of being on sick leave due to LPP. There was a trend of reduced risk of sick leave when allocated to the exercise group in the RCT.

### Strengths and weaknesses of the study

Strengths of the present study are the large numbers of participants, the prospective data collection and the secondary analyses of data from an RCT. A possible weakness was that information about LPP and sick leave were based on self-reports, and this may lead to bias with respect to location of the pain, duration of and reasons for sick leave. However, potential recall bias may have been reduced by the prospective design. It has been shown that self-reported sick leave data are valid, compared with data registered by insurance offices [17–19]. Furthermore, self-reported data are frequently used, but the sensitivity and specificity of the questions used in the present study are not known. Women were asked if they had been on sick leave since inclusion, and then asked to give information about the cause of their sick leave, dates for the sick leave period and also degree of sick leave. However, data regarding dates and degree of sick leave were too incomplete to be used. Information about degree (full-time or part-time) and length of sick leave could have increased the importance of the manuscript. To be able to get better information on this matter, future studies should perhaps use data from National registers on sick leave. Women participated voluntarily after receiving information about the study together with the invitation to the routine ultrasound scan. Therefore we do not have any information about the decliners. However, we found our sample comparable with the Norwegian mother and child cohort study (MoBa) regarding BMI and exercise frequency [12]. Further, only healthy women with singleton pregnancies were included and generalizing the results to other groups of pregnant women should be done with caution.

### Findings in relation to other studies

We found that work situations where it is not possible to make job adjustments was a risk factor for sick leave due to LPP in late pregnancy. No other studies exploring associations between pregnancy related sick leave due to LPP and job adjustments were found. There are, however, some available studies exploring sick leave in general and the need for job adjustments with results in accordance with our findings [4,20,21]. Sick leave was markedly less frequent when adjustment was not considered necessary and highest when adjustment was needed but not obtained [21]. Furthermore, women in need for, but not able to obtain adjustments had the highest sick leave rate [20]. Due to methodological differences further comparisons between the studies are difficult.

Two large Scandinavian birth cohorts have shown that occupational exposures are associated with an increased risk of sick leave [22], and that the necessity for job adjustment depends strongly on educational level, hectic pace and physical load at work [21]. The two previous studies have reported on sick leave in general, while our study has studied sick leave due to LPP in particular. Interestingly, we found that work schedule, working walking/standing, and whether women were able to decide working tasks and tempo themselves, were not associated with being on sick leave due to LPP. However, women in the present study reported on their work characteristics in mid-pregnancy. We have no information on work characteristics pre-pregnancy. It might be that highly exposed women already have had some job adjustments and thereby underestimated true associations. Being sick listed due to LPP at inclusion was found to be a risk factor. However, the confidence intervals were large, probably due to the low number on sick leave and also the fact that almost every woman on sick leave in early pregnancy was still sick listed in late pregnancy.

Pain location was a risk factor for being on sick leave in late pregnancy in the multivariable analyses. Surprisingly, having pain located in any part of the pelvic area increased the risk for being on sick leave due to LPP with almost the same OR (2.2 ≤ OR ≤ 2.7). This might be because we controlled for disability (DRI) in the model. By presenting a model 2 without DRI the OR’s for all the pain locations increased, and the largest risk for being on sick leave was with combined anterior and posterior pain (OR 11.0). These two models imply that both DRI and pain location in the pelvic area contribute independently into the model (model 1). Our findings are in accordance with previous studies that have showed that women with combined anterior and posterior pelvic pain have most reduced function and worst prognosis [13], and women with combined anterior and posterior pelvic girdle pain are more afflicted than women with anterior pain only or LBP [14]. Furthermore, one Scandinavian study combining data from three cohorts showed that it was the most afflicted women (with the most reduced physical function) that was on sick leave [23]. The present study support these findings with lower physical function, as measured with higher DRI, associated with need for sick leave due to LPP, and a strong association between pain location in the pelvic girdle and the need for sick leave due to LPP.

Allocation to the exercise group had a modest effect on reducing the risk for sick leave due to LPP in late pregnancy in the multivariable analysis. One recent meta-analysis including 11 RCTs found that exercise during pregnancy reduced the risk of sick leave due to LPP with 20% [24]. Further, a recent review found a modest effect of exercise during pregnancy to reduce LPP intensity, disability and sick leave [25]. Common for the studies reporting the best effects was that structured patient education was included in the intervention [25]. In general, adherence to the exercise protocols were low and the true effect may have been underestimated [24,25]. In the present study, only 55% of women in the exercise group adhered to the exercise protocol in the original RCT. The exercise program included exercises to strengthen large muscle groups, body awareness and lifting technique. Women in the exercise group received both written information about LPP as well as unstructured verbal information from the physiotherapists in charge of the exercise groups, about anatomy, pregnancy related changes and ergonomics. Pregnant women are potentially more vulnerable to musculoskeletal injuries due to the combination of increased joint laxity and increased weight gain with a shift in the point of gravity [26]. We therefore emphasized the importance of body awareness and lifting techniques in the verbal information. Although women in the exercise group reported to have exercised less than recommended, it could be that receiving the unstructured information may have been protective and thereby reduced the need for sick leave.

The two large Scandinavian birth cohort studies have reported that physical activity reduced PGP [27,28], and that engagement in physical exercise was associated with lower risk of sick leave in general in a dose-dependent way [29]. The current guidelines encourage healthy pregnant women to engage in regular exercise during pregnancy to promote health benefits [26]. Our findings might give an extra argument for recommending physical activity and exercise in pregnancy. Our multivariable regression analysis showed that being allocated to the intervention group had a modest association with reduced use of sick leave due to LPP, also when adjusting for other factors. Hence, the results can be seen to strengthen the evidence for benefits of physical exercise. However, strategies to increase adherence need to be studied.

### Meaning of the study

We have identified modifiable risk factors associated with sick leave due to LPP in late pregnancy. Facilitating job adjustment early on in the pregnancy, in cooperation with the employer, might be a precaution to keep more pregnant women in employment during pregnancy. Whether there is a causal relation must be addressed in future research. Further research is needed to explore how pregnant women can combine a modern work life with family life and a healthy lifestyle.
